# AGING IN PLACE: TURNING TO THE VOICES OF EXTENSION EDUCATORS

**DOI:** 10.1093/geroni/igac059.1994

**Published:** 2022-12-20

**Authors:** Susan Koerner, Brianna Anderson, Sehyun Ju

**Affiliations:** University of Illinois - Urbana-Champaign, Urbana, Illinois, United States; Saint Louis University, St. Louis, Missouri, United States; University of Illinois - Urbana-Champaign, Urbana, Illinois, United States

## Abstract

Most adults report a preference for aging-in-place (AIP) – remaining safely in their own home and community as they age, even as they become more dependent on others. When attempting to determine options and feasibility for AIP, older adults and/or their families – especially those living in non-metropolitan rural areas and small towns, may turn to Extension educators for information and guidance. For the current study we interviewed seven family-focused Extension educators responsible for 25 counties throughout a Midwestern state to explore the challenges, supports, patterns of experience, and service/policy recommendations that these professionals find relevant to AIP in their regions. The principal investigator (PI) conducted each semi-structured interview by phone; each audio-recorded interview lasted approximately 60 minutes. Two trained research assistants and the PI applied combined deductive-inductive thematic analyses to the transcribed interview data following Braun and Clarke (2012), utilizing MAXQDA, and ensuring trustworthiness during the coding process. Five major categories with sub-themes emerged: Challenges to AIP (e.g., transportation), Supports to AIP (e.g., churches), Most-Challenged Populations (e.g., middle-income families who neither can afford in-home assistance nor are eligible for government aid), Attitudes Toward AIP (e.g., caution against social isolation), and Recommendations for Services/Policies to Facilitate AIP (e.g., government funding, in-home technology assistance). Some variation across counties was apparent with, for example, one county making concerted efforts to retain young adults in its communities (i.e., reducing out-migration), thus enhancing family presence making AIP more feasible, non-isolating. Our findings will be especially noteworthy to social service providers and aging-focused public policymakers.

